# Green In Situ Synthesis of Silver Nanoparticles-Peptide Hydrogel Composites: Investigation of Their Antibacterial Activities

**DOI:** 10.3390/gels8110700

**Published:** 2022-10-29

**Authors:** Roya Binaymotlagh, Alessandra Del Giudice, Silvano Mignardi, Francesco Amato, Andrea Giacomo Marrani, Francesca Sivori, Ilaria Cavallo, Enea Gino Di Domenico, Cleofe Palocci, Laura Chronopoulou

**Affiliations:** 1 Department of Chemistry, Sapienza University of Rome, Piazzale Aldo Moro 5, 00185 Rome, Italy; 2 Department of Earth Sciences, Sapienza University of Rome, Piazzale Aldo Moro 5, 00185 Rome, Italy; 3 Microbiology and Virology, IRCCS San Gallicano Institute, via E. Chianesi, 53, 00144 Rome, Italy; 4 Department of Biology and Biotechnology “C. Darwin”, Sapienza University of Rome, Piazzale Aldo Moro 5, 00185 Rome, Italy; 5 Research Center for Applied Sciences to the Safeguard of Environment and Cultural Heritage (CIABC), Sapienza University of Rome, Piazzale Aldo Moro 5, 00185 Rome, Italy

**Keywords:** peptide-based hydrogels, biosynthesis, silver nanoparticles, antibacterial properties, hydrogel composites, *Staphylococcus aureus*

## Abstract

The present paper investigated the synthesis of peptide-based hydrogel composites containing photo-generated silver nanoparticles (AgNPs) obtained in the presence and absence of honey as tensile strength enhancer and hydrogel stabilizer. Fmoc-Phe and diphenylalanine (Phe_2_) were used as starting reagents for the hydrogelator synthesis via an enzymatic method. In particular, we developed an in situ one-pot approach for preparing AgNPs inside peptide hydrogels using a photochemical synthesis, without any toxic reducing agents, with reaction yields up to 30%. The structure and morphology of the nanohybrids were characterized with different techniques such as FESEM, UV-Vis, DLS, SAXS and XPS. Moreover, the antibacterial activity of these hybrid biomaterials was investigated on a laboratory strain and on a clinical isolate of *Staphylococcus aureus*. Results demonstrated that honey increased both swelling ability and also mechanical stability of the hydrogel. Finally, a higher antibacterial effect of AgNPs in the hybrid was observed in the presence of honey. In particular, AgNPs/hgel and AgNPs/hgel-honey showed an enhanced antibacterial activity (3.12 mg/L) compared to the free form of AgNPs, alone or in combination with honey (6.25 mg/L) for both *S. aureus* strains.

## 1. Introduction

The insurgence of resistance to multiple antimicrobial agents in pathogenic bacteria is already a major medical concern, compromising the clinical outcomes of many currently used drugs. By 2050, it is predicted that multidrug-resistant (MDR) pathogens will be causing more than 10 million deaths every year [1,2,3,4,5,6]. Unfortunately, the insurgence of MDR phenomena has overcome the rate of development of new antibiotics; therefore, exploring efficient or even alternative therapies seems urgent and necessary [7].

One of the most successful approaches against MDR is using nano-based organic or inorganic antibacterial agents, that may exert different or even more efficient antibacterial properties compared to traditional antibiotics [8,9]. Among inorganic nanostructures, the most promising candidates are silver nanoparticles (AgNPs), which demonstrate a potent and broad-spectrum efficacy in managing infections caused by multidrug-susceptible and multidrug-resistant pathogens like extended-spectrum beta-lactamase (ESBL)-producing *Escherichia coli*, methicillin-resistant *Staphylococcus aureus* (MRSA), and *Pseudomonas aeruginosa* [10,11,12,13]. In particular, silver sulfadiazine (SSD) can efficiently replace conventional antimicrobials, especially if used topically in burns and slow-healing wounds directly at the site of infection [14]. Considering several mechanisms of MDR, such as efflux pump and biofilm formation, AgNPs show a broad-spectrum antimicrobial activity that is superior to those of commonly used antibiotics [4,15]. As is well-known, four different mechanisms responsible for AgNPs antimicrobial activity have been identified, including (1) penetration inside bacteria damaging DNA or proteins, (2) disruption and permeabilization of bacterial cell walls, (3) generation of reactive oxygen species (ROS), and (4) penetration and intracellular damage disrupting metabolic pathways [9]. 

Another advantage of AgNPs is the availability of cost-effective synthetic methods for their preparation, including either chemical or physical approaches [4]. However, there is a growing interest in finding green reducing agents for the reduction of metallic ions such as silver in order to avoid remaining chemicals in the final products that may affect their biological applications. One green reducing agent can be sunlight, which is non-polluting, nontoxic and traceless in chemical processes [16,17,18]. To enhance the biological activity of AgNPs, these should be chemically stabilized by organic or inorganic species. In detail, this stabilization decreases the surface energy of bare AgNPs and protects the nanosurface against agglomeration and oxidation. Moreover, in biological applications of AgNPs, their bioavailability is controlled to have the maximum therapeutic effects. To achieve these features, encapsulation of AgNPs inside biocompatible materials may be developed as an alternative strategy for delivering antimicrobials.

Among biomaterials, peptide hydrogels have recently attracted growing interest for different applications, thanks to important features such as high water content, biocompatibility, biodegradability and possibility of tuning their microporous structures. Moreover, robust methods that allow hydrogel functionalization with a wide number of bioactive molecules, as well as the ability to incorporate different nanomaterials inside hydrogels, enabling the design of multi-functional platforms to fabricate advanced materials and composites have been investigated [19,20,21,22]. Apart from the above-mentioned features, some peptide hydrogels possess intrinsic antimicrobial and anti-inflammatory properties that may provide a synergistic effect when impregnated with antibiotics or nano-based antimicrobials [23].

In recent years, hydrogel-AgNPs composites have attracted a growing interest for antibacterial applications, because they benefit both properties of AgNPs and hydrogels in a single platform [24,25,26,27,28]. There are some reports for synthesizing short self-assembling peptides containing aromatic moieties in which AgNPs were successfully synthesized and encapsulated within the hydrogel by photochemical synthesis. Manisit Das et al. prepared a Phe-gly-gallol conjugate as a redox-active peptide to prepare AgNPs in the presence of sunlight with a size range between 10 and 20 nm using a fast one-pot method [29]. In 2010, Adhikari and Banerjee used the Fmoc-Val-Asp-OH dipeptide hydrogel as a template for the generation of Ag and Au nanoclusters by exploiting sunlight. They also showed that the synthesized composite is very stable even after 6 months at 4 °C [30]. In another study, Arab et al. used a tetrameric peptide able to self-assemble into a nanofibrous hydrogel resembling collagen properties. They synthesized AgNPs inside the hydrogel using UV irradiation [31]. 

**Scheme 1:**
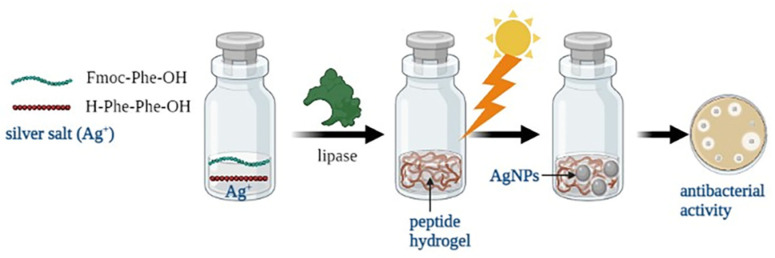
Illustration of in situ synthesis of AgNPs within peptide hydrogels using sunlight.

In this paper, we developed an in situ one-pot approach for preparing AgNPs inside peptide hydrogels using a photochemical synthesis, without any toxic reducing agents (Scheme 1). According to our findings, there is only one report that used this method to synthesize AgNPs inside a dipeptide hydrogel [30]. We biosynthesized a very short self-assembling peptide containing only three amino acids with the maximum aromatic moieties that a tripeptide can have, which is able to self-assemble in water. *Pseudomonas fluorescence* lipase was used to catalyze the synthesis of the Fmoc-Phe_3_ hydrogelator. The use of biocompatible peptide molecules, instead of toxic precursors, paves the way for an eco-friendlier generation of AgNPs. Moreover, in this study, honey was used to provide homogenized NPs with uniform and decreased size. Additionally, it was used to increase the tensile strength and mechanical properties of the hydrogel matrix. Honey is a carbohydrate-rich syrup that possesses interesting properties, such as antibacterial, antioxidant and anti-inflammatory features. In particular, honey antibacterial properties are correlated to its high osmolarity, pH, hydrogen peroxide production and presence of a variety of phytochemicals deriving from the plant’s nectar. It has been reported that in hydrogels preparation the incorporation of honey is able to enhance their tensile strength [32]. 

## 2. Results and Discussion

### 2.1. Preparation of AgNPs/Hydrogel Composites

**Figure 1:**
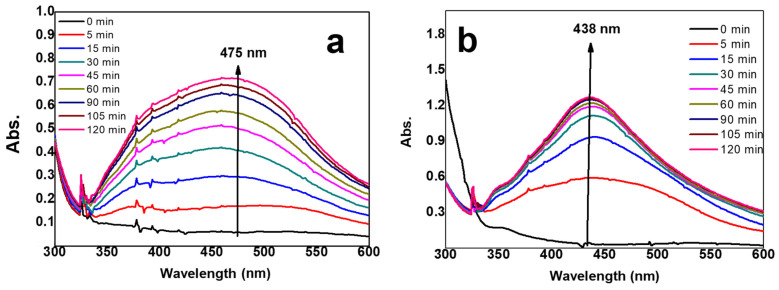
UV-Vis spectra of (**a**) AgNPs/hgel; (**b**) AgNPs/hgel-honey prepared at different irradiation times under sunlight exposure.

In our previous works, we have explored the possibility of preparing hydrogel composites based on short self-assembling peptides, incorporating different types of nanofillers or bioactive molecules to provide specific features [20,21,22]. In this work, we attempted to prepare a similar composite system, containing AgNPs, with antibacterial properties. On the basis of protocols optimized in previous works, we focused on developing a one-pot synthesis that could simultaneously allow the lipase-triggered formation of FmocPhe_3_ hydrogelator as well as the light-assisted reduction of Ag^+^ ions into AgNPs. We were able to observe successful hydrogel formation after incubation at 30 °C for about 30 min. For the formation of AgNPs that are accompanied by a change in color of the hydrogel containing them, light exposure was necessary. To investigate the effect of honey on composites, two different sets of experiments were performed, in the presence and absence of honey. To this aim, UV-Vis spectroscopy was used to study the plasmon band of in situ prepared AgNPs at different sunlight exposure times, with or without honey. As can be seen in Figure 1a, the light-assisted reduction of Ag^+^ ions inside the hydrogel results in a broad plasmon band centered around 475 nm, indicating the presence of large and heterogeneous particles, ascribable to the absence of any stabilizer in the reaction medium. On the other side, the second experiment, carried out in the presence of honey, exhibits a significant 37 nm blue-shift with respect to the first experiment (Figure 1b). This indicates the strong size quenching effect of honey for the AgNPs/hgel system. For both results, spectra reveal that the intensity of the absorption peaks increases with the exposure time. More importantly, Figure 1b demonstrates that the rate of AgNPs formation is increased by the presence of honey probably due to the presence of mild reducing agents in honey such as sugars [33].

The formation of AgNPs in the presence of sunlight might be due to the fact that the aromatic residues of the peptide may allow radical generation, since they absorb UV light at 257 nm, which is similar to the wavelength of irradiation light. This hints at the fact that UV irradiation of the peptide may generate radicals that are necessary for Ag^+^ reduction [4]. Moreover, after approximately 90 min, the intensity reaches a plateau, suggesting that NPs formation has completed.

### 2.2. Inductively Coupled Plasma-Atomic Emission Spectrometry (ICP-OES) 

In order to quantify the reduction of Ag^+^ ions and Ag^0^ formation, we analyzed samples with ICP-OES. Theoretically, there would be 20.00 ppm of Ag^0^ inside the sample solutions if the sunlight required by our protocol could successfully reduce the entire amount of Ag^+^ precursors used in the synthesis. However, the results of ICP-OES showed the presence of only 5.91 ± 0.12 ppm of silver inside the diluted solutions. Since the unreacted silver ions had been filtered out of the suspensions before the measurements, we calculated the reaction yield for the formation of AgNPs, by dividing the theoretical value to the obtained value from ICP-OES measurements, which was 29.5%. This suggests that the use of honey does not have any significant effect on NPs yield (data not shown).

### 2.3. Dynamic Light Scattering (DLS)

**Figure 2:**
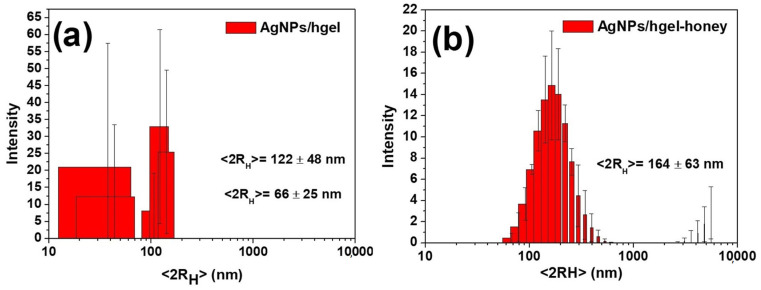
Hydrodynamic diameter distributions of (**a**) AgNPs/hgel; (**b**) AgNPs/hgel-honey measured by DLS.

Dynamic light scattering measurements were carried out to measure the size and degree of poly-dispersion of AgNPs produced with and without the honey. Figure 2a shows the size distribution of the AgNPs/hgel system, supporting the presence of two populations in this experiment with an average diameter of 66 ± 25 and 122 ± 48 nm for AgNPs in these conditions. This is consistent with the UV-Vis spectra obtained, which showed a broad plasmon band supporting the observed poly-dispersity (see previous section, Figure 1). However, in the presence of honey (Figure 2b), the particles dimensions are more homogeneous compared to the first system, and only one population is observed in these conditions, with a mean particle size of 164 ± 63 nm and a PDI of 0.3 demonstrating monodispersed AgNPs, confirming the stabilizing effect of honey on the size of AgNPs, which is consistent with the UV-Vis data.

### 2.4. SEM Measurement

**Figure 3:**
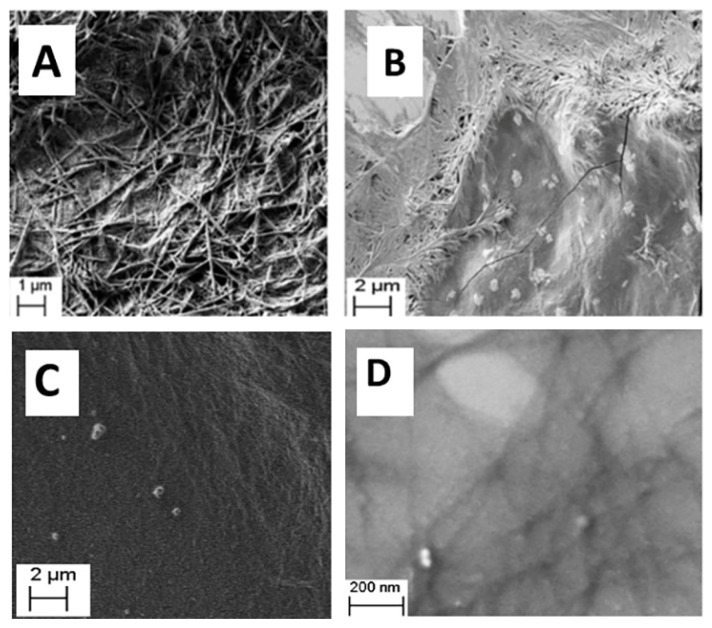
FESEM images of (**A**): hgel; (**B**): AgNPs/hgel; (**C**,**D**): AgNPs/hgel-honey.

The morphology of the hgel, AgNPs/hgel and AgNPs/hgel-honey was investigated with the SEM technique. Figure 3A shows the three-dimensional fibrillar network of the hgel. The length of the fibrils constituting the network is several micrometers and their width is 100–150 nm. On the other hand, SEM micrographs reported in Figure 3B,C show the AgNPs/hgel and AgNPs/hgel-honey samples. The presence of AgNPs covered by fibers is evident, suggesting the incorporation of AgNPs within the hydrogel fibrillar network in both samples. Regarding the SEM images obtained for AgNPs in the absence and presence of honey (Figure 3B–D respectively), greater aggregation is shown when the synthesis is carried out without honey. It can be deduced that honey itself would increase the degree of stabilization and particle size reduction of AgNPs.

### 2.5. X-ray Photoelectron Spectroscopy (XPS)

**Figure 4:**
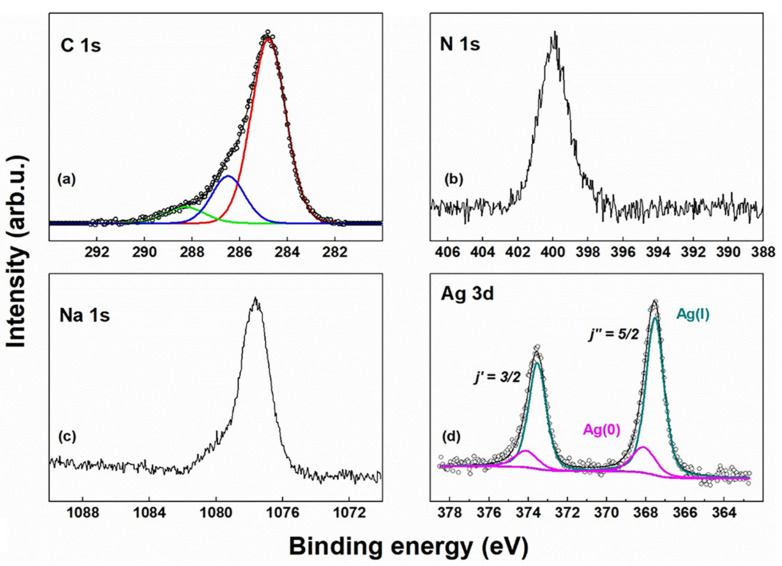
XPS spectra of Fmoc-(L-Phe)_3_ hydrogel + Ag^+^ in the regions of (**a**) C 1s, (**b**) N 1s, (**c**) Na 1s, (**d**) Ag 3d.

To further investigate the nature of Ag species within the hydrogel, XPS analyses were conducted on the pristine hydrogel (hgel) (control experiment, not shown) and on the AgNPs/hgel sample obtained from photochemical reduction of AgNO_3_, with the spectra of the latter shown in Figure 4. In spectrum (a) of Figure 4, the C 1s photoionization region is reported, which shows three main contributions attributed to the more representative chemically inequivalent C atoms in the Fmoc-(L-Phe)_3_ backbone: (i) aromatic and aliphatic C atoms at 284.8 eV (red curve); (ii) C atoms at 286.5 eV, presumably associated to the -NH-C(H)-C(O)-NH- fragment; (iii) amidic C in the -C(O)-NH- fragment at 288.2 eV, with small contributions from -C(O)O- (carboxylate) and -NH-C(O)O- (carbamate) fragments [34]. The N 1s spectrum (Figure 4b) shows a single contribution at 399.9 eV, perfectly compatible with an amidic N atom [35], while possible residuals of NO_3_^-^ can be excluded. The presence of Na was also detected (Figure 4c), yet the Na 1s electrons displayed a binding energy of 1077 eV, higher than that usually detected in sodium salts (~1072 eV) [34]. This shift may stem from possible aggregates of NaCl resulting from the NaOH/HCl treatment during preparation, which induce a partial electrostatic charging under the X-ray beam. The presence of AgNPs was ascertained by recording the photoelectron spectrum in the region of Ag 3d (Figure 4d). In this case, a spin-orbit split doublet appears (ΔE_so_ = 6.0 eV) with components in the area ratio 6:4, as expected for *j″* = 5/2 and *j′* = 3/2 total angular momentum values. According to peak-fitting results, two chemical components contribute to this signal, a predominant one at 367.5 eV and another at 368.1 eV (*j″* = 5/2 components). These features can be assigned to Ag(I) (dark cyan curve in Figure 4d) and Ag(0) (magenta curve in Figure 4d), respectively, in accordance with the literature [35]. The presence of a majority Ag(I) component calls for the formation of a shell of oxidized silver around the metal core of the NPs. The low intensity of Ag metal signal is probably due to the attenuation of electrons from the inner core of the NP, while the outer shell signal is enhanced.

### 2.6. X-ray Small Angle Scattering (SAXS)

**Figure 5:**
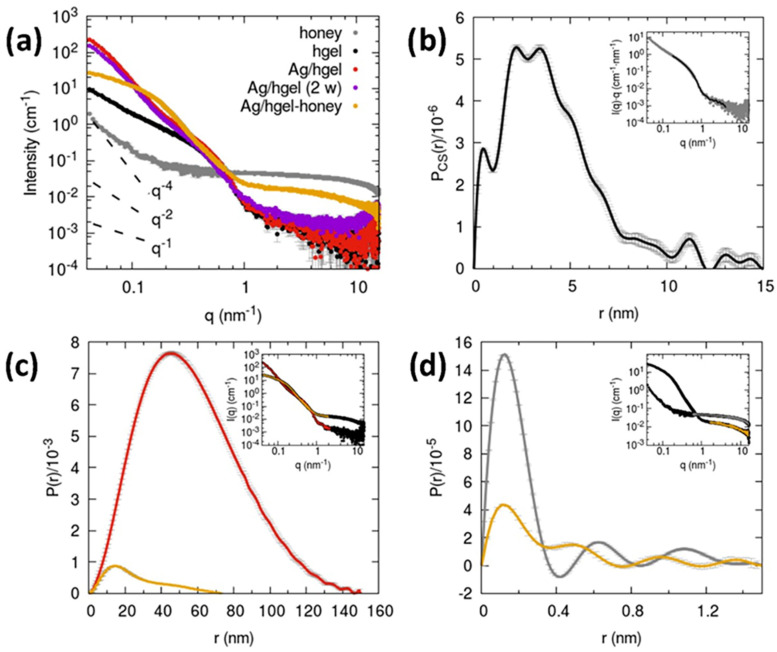
(**a**) SAXS profiles of honey, hgel, AgNPs/hgel, and AgNPs/hgel-honey. Characteristic power laws are reported for reference; (**b**) pair distance distribution function of the cross section P_CS_(r) obtained for the hgel sample (fit in the inset). (**c**) Pair distance distribution functions P(r) obtained for the Ag/hgel and Ag/hgel-honey samples (fits in the inset). (**d**) P(r) obtained for the honey and Ag/hgel-honey samples considering the high q portion of the data (q > 1 nm^−1^) (fits in the inset).

To study the structural features of hgel, AgNPs/hgel, and AgNPs/hgel-honey, the SAXS profiles for these three samples were collected. For the AgNPs/hgel system, two replicates were analyzed, and one sample was prepared two weeks before the analysis. As can be seen in Figure 5, the data of the AgNPs/hgel (1) (red) and the AgNPs/hgel (2 w) (purple) show overall the same profile indicating a good stability over time and reproducibility of the process of sample preparation, leading to the same structure at the investigated length-scales (1–100 nm). 

**Figure 6:**
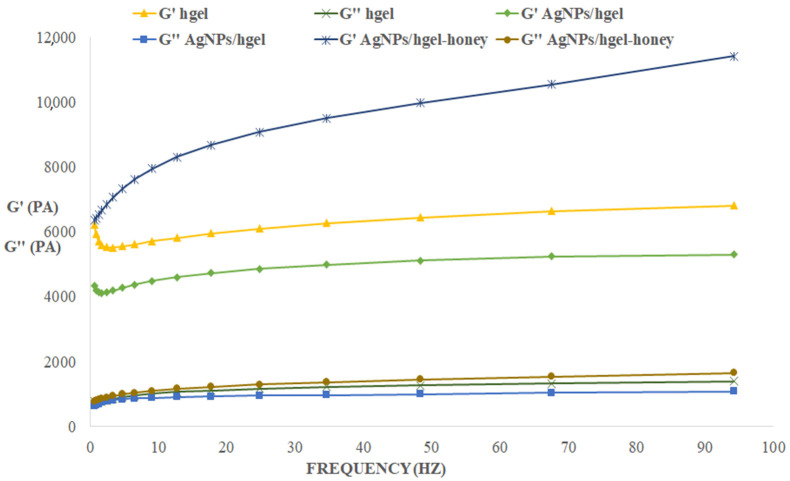
Frequency sweep of hgel, AgNPs/hgel and AgNPs/hgel-honey samples.

We can mention that the visual appearance of the samples results slightly inhomogeneous at a macroscopic scale, showing regions with a more intense red color probably related to a higher local concentration of AgNPs; therefore, for SAXS data collection we tried to sample the regions of capillaries with an intense color. All samples show isotropic scattering patterns even if the pre-formed gels were injected into borosilicate capillaries with a long needle syringe and then shaken to fill the bottom. The data of the pristine hydrogel (hgel, black dots) show the profile of fibrillar structures [20] having a cross-section with a radius of gyration of approximately 3 nm and an overall maximum diameter of around 8–10 nm as also deduced by the pair distance distribution function (Figure 6b). For *q* < 0.2 nm^−^^1^ the slope deviates from the *q*^−^^1^ predicted for rod-like objects towards more negative values (towards −2) and this could be interpreted as due to the branched fibrillar network (the fibrils cannot be seen as individual rods anymore at larger length scales compared to their cross-sections). The SAXS profile obtained for the AgNPs/hgel is in the *q*-range > 0.4 nm^−^^1^ almost superimposable to that of the hydrogel sample, suggesting that the fibrillar cross section is not perturbed. However, additional scattering signal and oscillations can be seen in the lower *q*. We could attribute these to the Ag nanoprecipitated structures within the hydrogel and considering the impossibility of seeing a Guinier region for these inhomogeneities we could estimate that their size is above 100 nm.

For the AgNPs/hgel obtained in the presence of honey, the SAXS curve shows marked differences from the AgNPs/hgel sample both in the low *q* (<0.4 nm^−^^1^) and the higher *q* (>1 nm^−^^1^); in the latter region a signal related to some inhomogeneities with a radius of gyration smaller than 0.2 nm is detected. We hypothesize this high *q* scattering signal comes from honey molecular components like small oligosaccharides, since a sample of diluted honey at the same concentration used for the AgNPs/hgel synthesis also shows a similar contribution, corresponding to even smaller sizes (overall < 0.4 nm, Figure 6d). Regarding the shape of the SAXS profile in the low *q*, we can comment that for the AgNPs/hgel-honey sample it shows a lower slope and much lower intensity than the AgNPs/hgel sample, suggesting smaller structures (diameters of approximately 30 nm) are formed by the Ag nanoprecipitation in the presence of honey (Figure 6c). We could relate this observed difference to the difference in visual appearance between the Ag-gels obtained without and with honey (opaque with larger precipitates vs. transparent with smaller precipitates), which are related to light scattering phenomena, the UV-Vis absorption data presented in Section 2.1, and the SEM micrographs in Section 2.4.

### 2.7. Rheological Studies

The rheological properties of hydrogel materials play a pivotal role in defining their application potential, so their evaluation is of crucial importance. The goal of this analysis is to understand how the presence of AgNPs synthesized in situ affects the viscoelastic behavior of the hydrogels, by measuring G’ (elastic modulus) and G” (viscous modulus). As shown in Figure 6, the AgNP/hgel had the lowest storage modulus. These results suggested that incorporating AgNPs inside the matrix decreased mechanical strength. Regarding the AgNPs/hgel-honey composite, there is a significant increase in both G’ and G” moduli and therefore a marked improvement in mechanical properties can be seen. The complex biological matrix of honey, which acts as a stabilizer for AgNPs, may itself interact with the peptide components in the hydrogel, forming further crosslinks and consequently making the gel stronger.

### 2.8. Swelling Ability

The swelling ability of AgNPs/hgel and AgNPs/hgel-honey samples was measured and compared with that of the hgel (Table 1). The results reveal that AgNPs/hgel showed higher swelling behavior in comparison to the hgel. The enhancement in swelling behavior of AgNPs/hgel might be related to the presence of AgNPs in different sizes and surface charges, that resulted in the penetration of a higher amount of water molecules in order to counterbalance the built up osmotic pressure [15]. However, this swelling enhancement also might be related to the formation of AgNPs inside of the hgel matrix that expand the hydrogel network and consequent more water absorption [15]. Regarding the AgNPs/hgel-honey, it increased the swelling behavior the most which is related to its hygroscopic features [36]. 

### 2.9. Antibacterial Studies

**Figure 7:**
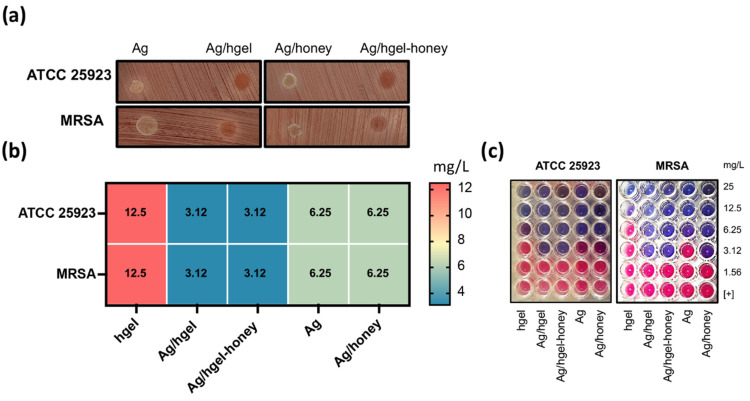
Antimicrobial susceptibility testing (AST) of AgNPs in the hydrogel composite compared to the free form of AgNPs against *Staphylococcus aureus* ATCC 25923 and the methicillin-resistant *Staphylococcus aureus* (MRSA) clinical isolate. (**a**) Representative pictures for the plate inhibition zone test performed for different AgNPs formulations against ATCC 25923 and MRSA. Chocolate agar plates contained 20 µL of 3.12 mg/L solution of each compound tested. (**b**) Minimum inhibitory concentration (MIC) for hgel, AgNPs/hgel (Ag/hgel), AgNPs/hgel-honey (Ag/hgel-honey), AgNPs alone (Ag) and AgNPs + honey (Ag/honey). (**c**) Determination of MIC by resazurin microtiter assay method for the *Staphylococcus aureus* ATCC 25923 strain. Bacterial cells, incubated in cation-adjusted Mueller-Hinton broth (MHB) in the presence of resazurin (blue), were exposed to different compounds for 24 h. A change from blue to pink is indicative of the reduction of resazurin and, consequently, bacterial growth. The MIC was defined as the lowest drug concentration that prevented this color change. Experiments were performed in triplicate and repeated three times.

The antimicrobial susceptibility profiles of the laboratory strain *S. aureus* ATCC 25923 and MRSA clinical isolate for the tested compounds are summarized in Figure 7. In particular, the hydrogel alone showed a reduced toxicity compared to AgNPs and AgNPs-honey, with a minimum inhibitory concentration (MIC) of 12.5 mg/L for both *S. aureus* ATCC 25923 and the MRSA isolate. Conversely, the AgNPs/hgel and AgNPs/hgel-honey showed an enhanced antibacterial activity (3.12 mg/L) compared to the free form of AgNPs, alone or in combination with honey (6.25 mg/L) for both *S. aureus* ATCC 25923 and the MRSA isolate.

## 3. Conclusions

In this work, we prepared highly aromatic tripeptide hydrogel composites containing AgNPs using a one-pot green synthetic method. AgNPs were photosynthesized inside peptide hydrogels in the presence or absence of honey, used as a green material for capping AgNPs, improving the mechanical properties of the hydrogel. The aromatic moieties of the peptide building blocks of the gel played a critical role in the reduction of silver salts, as they produced electron radicals by absorbing sunlight. We observed that the addition of honey to the composites not only decreased the particle size of AgNPs, producing homogeneous NPs, but it also improved the swelling ability and the mechanical properties of the gel (which are important features for future applications such as wound healing). The results of the antibacterial studies demonstrated that the AgNPs/hgel and AgNPs/hgel-honey showed significantly lowered MIC for both *S. aureus* ATCC 25923 and the MRSA isolate in comparison to hgel, Ag, and Ag/honey.

## 4. Materials and Methods

### 4.1. Materials

*N*-(9-Fluorenylmethoxycarbonyl)-L-phenylalanine (Fmoc-L-phenylalanine: Fmoc-Phe-OH, 99%, 387.44 g/mol) and L-Phenylalanyl-L-phenylalanine (H-Phe-Phe-OH, 98%, 312.36 g/mol) were purchased from Bachem GmbH (Weil am Rhein, Germany) and used as received. Commercially prepared Miele Millefiori (G.B. Ambrosoli SpA, Ronago, Italy) was used as received. Lipase from *Pseudomonas fluorescens* (PFL ≥ 20,000 U/mg), silver nitrate (AgNO_3_, 98%) and all other chemicals and solvents were obtained from Sigma Aldrich (St. Louis, MO, USA) and used as received.

### 4.2. Biosynthesis of Hydrogel Composites

FmocPhe_3_ hydrogel (hgel) preparation was carried out according to our previous works [20]. To prepare hydrogels impregnated with AgNPs (AgNPs/hgel), FmocPhe and Phe_2_ were added in equimolar quantities to a mixture containing 2 mL aqueous solution of AgNO_3_ (5 mM) and 420 µL of 0.5 M NaOH, stirring magnetically for 10 min. Then, pH was adjusted to 7 by adding 1.5 mL of 0.1 M HCl. Next, 100 µL lipase aqueous solution (50 mg/mL) was added and the reaction mixture was incubated at 30 °C for 30 min. After gel formation, samples were exposed to the sunlight for 1.5 h. In the case of AgNPs/hgel prepared in the presence of honey (AgNPs/hgel-honey), equimolar quantities of the amino acid and dipeptide along with 750 µL of H_2_O and 315 µL of NaOH were mixed and stirred for 10 min. Then, 333 µL of AgNO_3_ were then added to the mixture and the pH was decreased to around 7 using HCl. Before the addition of PFL solution, 900 µL of honey (350 mg/mL) was introduced and the mixture was incubated for 30 min at 30 °C in a thermostated bath.

### 4.3. UV-Vis Spectroscopy and Dynamic Light Scattering (DLS)

All UV-Vis spectra were recorded in 1.00 cm optical path quartz cells with a Cary 100 Varian spectrophotometer. Dynamic light scattering (DLS) measurements were performed using a Zetasizer Nano S (Malvern Instruments, Malvern, UK) equipped with a 4 mW He-Ne laser (633 nm), with a minimum of 10 replicates. All measurements were carried out a minimum of three times, reporting the average value ± standard deviation. Peak intensity analysis was used to calculate the average hydrodynamic diameter of the samples.

### 4.4. Electron Microscopy Studies

FESEM images were obtained using a variable pressure scanning electron microscope (VP-SEM, Hitachi SU-3500) equipped with dual-energy-dispersive X-ray spectroscopy detectors (VP-SEM-dEDS) in a parallel configuration (Bruker, XFlash 6|60) and a high active area (60 mm^2^ each). Samples were deposited onto stubs without the need of a conductive coating and analyzed at an accelerating voltage that avoided radiation damage.

### 4.5. X-ray Photoelectron Spectroscopy (XPS)

For XPS analysis, a modified Omicron NanoTechnology MXPS system was employed. Samples were excited by achromatic AlKα photons (hν = 1486.6 eV), operating the anode at 14–15 kV, 10–20 mA. The take-off angle and pass energy were fixed at 21° and 20 eV, respectively. Samples were prepared by casting onto a hydrogenated Si(100) wafer a 20 μL drop of a hydrogel where the initial Ag^+^ concentration was 15 mM. The obtained Si-supported sample was left to dry overnight, and then mounted on a stainless steel sample holder for measurement.

### 4.6. Inductively Coupled Plasma-Atomic Emission Spectrometry (ICP-AES)

ICP-AES was performed to find the yield of the in situ formed AgNPs after exposure. First, the nanoparticles were separated from unreacted silver ions using 3 kDa membrane filter (Amicon Ultra-100 Centrifugal Filter Unit, Millipore, UK) inside a centrifuge with 15,000 rpm for 30 min at 20 °C. Then the purified nanoparticles were digested in HNO_3_ overnight, and the final solution was diluted by a final factor of 25 before the measurements. The amount of formed AgNPs in ppm were analyzed by inductively coupled plasma atomic emission spectrometry (ICP-AES) with a Varian Vista RL CCD Simultaneous ICP-AES spectrometer. The analytical detection limit for Ag was 0.04 mg/L, and analytical errors were estimated to be in the order of 3% using Ag emission line 338.289 nm. The formation yield for AgNPs was calculated with the following equation:

$$yield=\frac{theoretical\;Ag\;quantity}{experimental\;Ag\;quantity}\times 100$$

### 4.7. Small Angle X-ray Scattering (SAXS)

The X-ray scattering measurements were conducted at SAXS Lab Sapienza with a Xeuss 2.0 Q Xoom system (Xenocs SA, Grenoble, France) equipped with a micro-focus Genix 3D X-ray Cu source and a two-dimensional Pilatus3 R 300 K detector (Dectris Ltd., Baden, Switzerland) as reported previously [20]. 

### 4.8. Rheological Measurements

To characterize the composites from a rheological point of view, it is possible to exploit dynamo-mechanical analysis (mechanical spectroscopy). In this case, the test in oscillatory mode was carried out on the hydrogels with an Anton Paar MCR 302 rotational rheometer, registering the elastic (*G*′) and viscous (*G*″) moduli trend, in the presence and absence of silver nanoparticles, varying the frequency of application of the stimulus and keeping the intensity of the deformation to which the samples are subjected constant (shear strain = 1%). These frequency sweep experiments are carried out with a rotational rheometer having a plate-plate geometry, keeping the gap between the two plates constant (1 mm). The test is performed under constant temperature conditions (30 °C).

### 4.9. Swelling Ability

After hydrogel formation, 3 mL of phosphate buffer solution (PBS, pH = 7.4) was added and incubated for 24 h at 30 °C in a thermostatic bath. Then PBS was removed, and the hydrogels were lyophilized. The swelling degree was calculated according to the equation below reported:

*q* = (*W*_s_ − *W*_d_)/*W*_d_ (1)

*q* = swelling degree, *W*_s_ = hydrogel weight after PBS removal, *W*_d_ = weight of lyophilized gel.

### 4.10. Test Microorganisms Used in This Study

*S. aureus* ATCC 25923 was purchased from the American Type Culture Collection. The methicillin-resistant *Staphylococcus aureus* (MRSA) clinical isolate was provided by the Microbial Strain Repository of the Microbiology and Virology laboratory of San Gallicano Dermatology Institute, Rome, Italy, collected in 2020 from a patient presenting skin and soft tissue infections [37].

### 4.11. Plate Inhibition Zone Assay to Test for Antimicrobial Activity

An overnight culture of *S. aureus* ATCC 25923 or MRSA grown on a blood agar plate was used to inoculate 2 mL of 0.45% saline solution to 0.5 ± 0.1 McFarland turbidity standard (~108 CFU/mL). Then, the bacteria were swabbed onto a Chocolate agar plate (bioMérieux, Marcy-l’Étoile, France). A 20 µL drop containing AgNPs formulations was released on the top of the agar plate and incubated at 37 °C for 24 h. A 0.45% saline solution was used as the positive control. After 24 h of incubation, the diameter of the inhibition zone was measured, and plates were photographed.

### 4.12. Determination of Minimal Inhibitory Concentration (MIC)

The experiments were conducted as previously described [38]. Antimicrobial susceptibility testing (AST) of silver nanoparticles in the hybrid in the free form was performed as described previously [39]. The metabolic activity of the treated planktonic bacterial cultures was evaluated in the presence of serial two-fold dilutions of different compounds [37]. Experiments were conducted in triplicate and repeated three times.

## Figures and Tables

**Table 1:** Swelling abilities of the hydrogel systems.

Samples	Swelling Degree (q)
hgel	62.18 ± 0.35
AgNPs/hgel	75.99 ± 0.65
AgNPs/hgel-honey	79.11 ± 0.23

## Data Availability

Data available on request.
